# Beyond Binary Positivity: Spectrum of Nodal Tumor Burden in Sentinel Lymph Node Biopsy for High-Risk Cutaneous Squamous Cell Carcinoma

**DOI:** 10.3390/dermatopathology13020020

**Published:** 2026-04-30

**Authors:** Irena Janković, Goran Stevanović, Toma Kovačević, Dimitrije Janković, Dimitrije Pavlović

**Affiliations:** 1 Clinic for Plastic and Reconstructive Surgery, University Clinical Center Niš, Bul. Dr Zorana Đinđića 48, 18000 Niš, Serbia; gste66@yahoo.com (G.S.); dimitrije.pavlovic@medfak.ni.ac.rs (D.P.); 2 Department of Surgery and Anesthesiology with Reanimatology, Faculty of Medicine, University of Niš, Bul. Dr Zorana Đinđića 81, 18000 Niš, Serbia; 3 Clinic for Otorhinolaryngology, University Clinical Center Niš, Bul. Dr Zorana Đinđića 48, 18000 Niš, Serbia; tomakovacevic1994@gmail.com; 4 Department of Otorhinolaryngology, Faculty of Medicine, University of Niš, Bul. Dr Zorana Đinđića 81, 18000 Niš, Serbia; 5 Apotekarska ustanova Niš (Public Pharmacy Institution Niš), Bul. Dr Zorana Đinđića 6, 18000 Niš, Serbia; jankovicdill@gmail.com

**Keywords:** cutaneous squamous cell carcinoma, sentinel lymph node biopsy, tumor burden, isolated tumor cells, micrometastasis, nodal staging, ultrastaging, skin cancer, high-risk cSCC

## Abstract

**Background and Objectives**: Sentinel lymph node biopsy (SLNB) is increasingly used for high-risk, clinically node-negative cutaneous squamous cell carcinoma (cSCC), yet pathological reporting remains binary, lacking morphological stratification. The prognostic relevance of nodal tumor burden subtypes—isolated tumor cells (ITC), micrometastases, and macrometastases—is well established in melanoma and breast cancer but remains uncharacterized in cSCC. We aimed to describe the morphological spectrum of sentinel lymph node involvement in a consecutive institutional cohort and determine whether primary tumor characteristics predict the extent of nodal colonization. **Materials and Methods**: We conducted a retrospective-observational study at Clinical Center Niš (Serbia) including 35 consecutive clinically N0 high-risk cSCC patients who underwent SLNB using a dual-tracer protocol (^99^mTc-labeled albumin and methylene blue). Sentinel nodes were processed by serial sectioning with hematoxylin-eosin and pancytokeratin (AE1/AE3) immunohistochemistry. Deposits were classified as ITC (≤0.2 mm), micrometastases (>0.2–2.0 mm), or macrometastases (>2.0 mm). Clinicopathologic predictors were evaluated using the Mann–Whitney U test, Fisher’s exact test, the Kruskal–Wallis test, and the Spearman rank correlation test. **Results**: SLN involvement was identified in 12 of 35 patients (34.3%). Among positive cases, ITC accounted for 6 patients (50.0%), micrometastases for 5 (41.7%), and macrometastasis for 1 (8.3%)—minimal nodal disease constituting 91.7% of positive findings. No primary tumor feature—including diameter, thickness, grade, perineural invasion, or lesion multiplicity—significantly distinguished ITC from overt metastatic deposits. Patients with ITC showed numerically higher median tumor thickness (8.0 mm) than those with micrometastases (4.0 mm), though this did not reach significance (Kruskal–Wallis *p* = 0.065). **Conclusions**: SLN positivity in high-risk cSCC is morphologically heterogeneous, with minimal nodal disease predominating. Primary tumor features do not reliably stratify the extent of nodal colonization. Structured tumor-burden reporting—distinguishing ITC, micrometastases, and macrometastases—should be adopted as standard practice to enable meaningful prognostic comparisons and inform individualized management.

## 1. Introduction

Cutaneous squamous cell carcinoma (cSCC) is the second most common malignancy in the human population and the most prevalent keratinocyte-derived cancer after basal cell carcinoma [1]. Its global incidence has been rising steadily for decades, driven by cumulative ultraviolet radiation exposure, an aging demographic, and expanding immunosuppressed patient populations [2,3]. In the United States alone, the annual incidence of invasive cSCC was estimated at approximately 700,000 cases in 2012, with disease-specific mortality approaching that of melanoma and renal cell carcinoma in some regions of the country [4]. In Europe, upward trends in age-standardized incidence rates have been documented across multiple national registries, with no evidence of near-term stabilization [2].

Although the vast majority of cSCCs are amenable to surgical cure with standard excision, a clinically important minority—estimated at approximately 2–5% of all cases—will develop regional lymph node metastasis, and up to 2% will die of the disease [4,5]. This subset represents a disproportionately large burden on healthcare resources and carries a substantially worse prognosis than node-negative disease. Historically, regional failure has been associated with five-year disease-specific survival rates below 50%, and the presence of clinically overt nodal metastasis at presentation is independently associated with distant dissemination [6]. The challenge, therefore, lies not in managing established nodal disease—which is relatively straightforward—but in accurately identifying, at the time of primary tumor excision, which clinically node-negative patients harbor occult regional metastases that would benefit from early intervention.

The concept of the sentinel lymph node (SLN)—the anatomically first node to receive lymphatic drainage from a primary tumor—was formally introduced into clinical practice by Morton and colleagues in 1992, initially in the management of melanoma [7]. The SLN concept is based on the observation that lymphatic metastasis follows an orderly, predictable anatomical pathway: if the sentinel node is tumor-free, the downstream nodal basin is overwhelmingly likely to be free of metastatic disease as well. Following validation across large multicenter trials and widespread adoption in melanoma and breast cancer, SLN biopsy (SLNB) has become one of the most important advances in surgical oncology of the past three decades [8]. Its central virtue is the substitution of a minimally invasive, targeted procedure for a morbid prophylactic lymphadenectomy in the large majority of patients who do not harbor nodal disease.

Application of SLNB to cSCC has developed more slowly and remains less standardized than in melanoma. Early series demonstrated technical feasibility and acceptable SLN identification rates, generally exceeding 95% with dual-tracer protocols combining radiocolloid and blue dye [9,10]. Subsequent systematic reviews and meta-analyses documented overall SLN positivity rates ranging from 12% to 21% across unselected cSCC series, with substantial heterogeneity attributable to differences in patient risk stratification, primary tumor site, and pathological processing protocols [9,11]. A systematic review and meta-analysis focusing specifically on head and neck cSCC—the predominant anatomical subtype in most European series—reported a pooled positivity rate of 5.6% with a high SLN identification rate of 98.8%, alongside a cumulative regional recurrence rate of 2.9% following a negative SLNB [12]. These data collectively support the technical reliability of the procedure in the cSCC context, yet also highlight that patient selection and risk stratification are the critical determinants of clinical utility.

High-risk cSCC is defined by the co-occurrence of adverse clinicopathologic features that collectively predict an elevated likelihood of regional and distant failure. The two most widely used staging systems—the American Joint Committee on Cancer 8th edition (AJCC-8) and the Brigham and Women’s Hospital (BWH) system—operationalize high-risk designation differently but share a core set of recognized risk factors: tumor diameter ≥ 2 cm, depth of invasion beyond the subcutaneous fat, perineural invasion (particularly of large-caliber nerves ≥0.1 mm), poor histological differentiation, and anatomically adverse locations (ear, lip, temple) [13,14]. The BWH system, which stratifies patients into four T-stages based on cumulative risk-factor count, has been validated as superior to AJCC-8 in discriminating patients at risk for nodal metastasis: T2b tumors (two or three high-risk features) carry a nodal metastasis risk of approximately 29%, and T3 tumors (all four features or bone invasion) exceed 50% in some series [14,15]. This risk gradient forms the conceptual rationale for selective SLNB in high-risk cSCC.

A critical but underappreciated determinant of the information yielded by SLNB is the pathological protocol used to examine the excised nodes. Standard single-section hematoxylin and eosin (H&E) processing, which remains common in non-melanoma skin cancer pathology, is insensitive to microscopic nodal deposits. The technique of ultrastaging—involving serial multi-level sectioning combined with immunohistochemical (IHC) staining for epithelial markers—substantially increases the detection rate of low-volume nodal disease [16,17]. Ultrastaging was first systematically applied in breast cancer, where it enabled formal recognition of ITC (≤0.2 mm) and micrometastases (>0.2–2.0 mm) as distinct categories with different prognostic implications, codified in the AJCC 6th edition in 2002 [18]. The application of analogous tiered protocols to cSCC SLNB has been inconsistent across published series, creating a fundamental comparability problem: studies that did not use ultrastaging almost certainly underdetect low-volume disease, making their positivity rates and prognostic conclusions unreliable for high-sensitivity settings.

In melanoma, the prognostic significance of tumor burden within the SLN has been extensively characterized. Nodal tumor burden—encompassing quantitative parameters such as maximum tumor deposit diameter, tumor penetrative depth, and location within the node (subcapsular versus parenchymal)—is an independent predictor of non-sentinel node involvement, distant recurrence, and melanoma-specific mortality, and forms a key component of contemporary melanoma staging [19,20]. The Rotterdam and Dewar classification systems have been prospectively validated and are integral to clinical decision-making regarding completion lymph node dissection. In breast cancer, the distinction between ITC, micrometastasis, and macrometastasis has direct therapeutic implications and is embedded in the AJCC staging system [18]. By contrast, no validated, consensus-based tumor burden classification system exists for cSCC, and current reporting practice remains predominantly binary—positive or negative, without quantitative or morphological stratification of the positive category [12].

This binary framing conflates biologically heterogeneous patterns of nodal colonization and limits comparability across institutions using different pathological protocols. No study to date has systematically characterized the internal morphological heterogeneity of the SLN-positive subgroup in cSCC. The present study addresses this gap by reporting the spectrum of nodal tumor burden in a consecutive institutional cohort of 35 high-risk, clinically node-negative cSCC patients managed with a standardized dual-tracer SLNB and ultrastaging protocol, and by assessing whether primary tumor characteristics predict the extent of nodal colonization.

## 2. Materials and Methods

### 2.1. Study Design and Setting

This retrospective-observational study was conducted at the Clinical Center Niš (Serbia), with participation of three institutional departments: the Clinic for Plastic and Reconstructive Surgery, the Institute of Nuclear Medicine, and the Institute of Pathology. We analyzed a consecutive series of patients with histologically confirmed high-risk cutaneous squamous cell carcinoma (cSCC) who underwent sentinel lymph node biopsy (SLNB) as part of institutional clinical practice. The study protocol was reviewed and approved by the Ethics Committee of the Faculty of Medicine, University of Niš (protocol number: 01-206-4, approved 18 January 2010). All patient data were anonymized prior to analysis in accordance with institutional data protection requirements.

### 2.2. Eligibility Criteria

Eligible participants were adults with clinically node-negative (cN0) high-risk cSCC and no evidence of regional nodal metastasis on clinical examination or preoperative ultrasound. High-risk cSCC was defined by the presence of one or more of the following features: anatomical location within the facial H-zone, auricle or periauricular region, scalp, dorsum of the hand, or sun-protected sites; primary tumor diameter greater than 2 cm; tumor thickness greater than 4 mm and/or higher Clark invasion level; ulceration; moderate-to-poor histological differentiation; clinical or histological immunodeficiency; perineural invasion; or tumor arising within previously damaged skin (scar, chronic ulcer, or radiation field). Patients with low-risk cSCC and those with clinically or sonographically evident regional nodal metastasis at the time of referral were excluded.

### 2.3. Surgical Procedure

In patients with clinical suspicion of cSCC, excisional biopsy was performed with approximately 5 mm clinical margins into healthy tissue, deliberately limited to preserve the integrity of lymphatic channels for subsequent mapping. Following histopathological confirmation of high-risk cSCC and confirmation of cN0 status, SLNB was scheduled as a second surgical procedure.

This staged approach—in which SLNB is performed as a separate, subsequent procedure rather than simultaneously with wide local excision—reflects a deliberate institutional protocol driven by diagnostic necessity. Unlike melanoma, where clinical and dermoscopic evaluation typically justifies SLNB planning before histological confirmation, cSCC frequently presents as a clinically ambiguous lesion, and its high-risk classification can only be established after full pathological assessment of the excision specimen. Scheduling SLNB only after histopathological confirmation of high-risk criteria ensures the procedure is limited to patients with a demonstrated indication, avoiding unnecessary intervention in patients whose lesions are low-risk on definitive pathology. Analogous staged approaches have been described in published cSCC SLNB series [10].

Sentinel lymph node identification employed a standardized dual-tracer technique combining a radiolabeled colloid and vital blue dye. Lymphoscintigraphy was performed after intradermal injection of 99mTc-labeled human serum albumin (particle size 200–600 nm; 50 MBq in 0.3 mL) using a two-day protocol. Dynamic anterior-projection imaging was performed until sentinel node visualization, followed by early static images; late static imaging was obtained on the day of surgery, approximately 16–18 h after tracer administration. On the operative day, 2 mL of 2% methylene blue solution was injected intradermally around the biopsy scar approximately 20 min before the procedure.

SLNB was performed under general anesthesia or monitored local anesthesia with sedation. Intraoperative sentinel node localization was achieved using a handheld gamma probe; nodes demonstrating radiotracer uptake and/or blue staining were excised. After nodal removal, the surgical bed was surveyed with the gamma probe to confirm residual counts ≤ 10% of the excised node activity; additional nodes were removed if this criterion was not met. Wide local excision of the primary tumor site was performed in the same operative session with oncologic margins (≥1 cm when anatomically feasible).

### 2.4. Pathological Processing of Sentinel Lymph Nodes

All excised sentinel lymph nodes and primary tumor specimens were fixed in 10% neutral-buffered formalin and embedded in paraffin. Sentinel nodes were processed according to a size-adapted sectioning protocol: nodes measuring less than 4 mm in maximum diameter were bivalved along the longitudinal axis and submitted in their entirety; larger nodes were serially sectioned at 2–3 mm intervals and submitted in their entirety. Each paraffin block was serially sectioned to yield three H&E-stained levels at 40–50 µm intervals; blank slides were reserved at each interval for immunohistochemical staining if required. When H&E sections were equivocal or negative but gross or microscopic features raised suspicion for metastatic involvement—including subcapsular sinusoidal distension, atypical single cells, or clusters not definitively identifiable as epithelial on H&E alone—immunohistochemistry (IHC) with a broad-spectrum pancytokeratin antibody (clone AE1/AE3, ready-to-use; DAB chromogen; hematoxylin counterstain) was performed on the reserved slides. This selective IHC protocol, applied to equivocal cases rather than universally, is consistent with ultrastaging approaches reported in the cSCC and melanoma SLNB literature. All six ITC cases in this cohort were confirmed by AE1/AE3 immunoreactivity.

Metastatic deposits identified within sentinel nodes were classified according to established size-based criteria, adapted from the framework originally codified for breast cancer sentinel node pathology: macrometastases were defined as deposits greater than 2.0 mm in maximum diameter; micrometastases as deposits greater than 0.2 mm and up to 2.0 mm; and isolated tumor cells (ITC)—also referred to as preclinical deposits (PC) throughout this report, reflecting the terminological practice of our institutional pathology unit—as deposits measuring 0.2 mm or less, or comprising fewer than 200 individual cells in a single histological cross-section. SLN status was considered positive if at least one node contained a metastatic deposit of any category. In cases where multiple discrete tumor deposits were identified within a single sentinel node, each deposit was measured individually; the patient’s nodal tumor-burden category was assigned based on the largest deposit present, consistent with the approach used in melanoma and breast cancer SLNB pathology.

Primary tumor thickness (Breslow equivalent, in millimeters) and histological grade (G1 through G3) were extracted from the primary tumor pathology report. Histological grade was additionally dichotomized as G1 versus G2–G3 (low versus high grade) for descriptive comparisons.

### 2.5. Unit of Analysis

Because multiple synchronous or metachronous lesions may originate from the same patient, all primary analyses were performed at the patient level. For patients with more than one lesion, the most aggressive tumor characteristics were used as representative values: maximum tumor thickness, highest histological grade, and largest tumor diameter.

### 2.6. Statistical Analysis

Continuous variables are presented as medians and interquartile ranges (IQRs); categorical variables are presented as counts and percentages. The primary comparisons—between the SLN-positive and SLN-negative subgroups across the full cohort—were performed using the Mann–Whitney U test for continuous variables and Fisher’s exact test for categorical variables. Statistical significance was defined as two-sided *p* < 0.05 throughout.

Within the SLN-positive subgroup (*n* = 12), clinicopathologic characteristics were compared between patients with isolated tumor cells (ITC/PC) and those with overt metastatic involvement (micrometastases and macrometastases combined) using the Mann–Whitney U test for continuous variables and Fisher’s exact test for categorical variables. This binary grouping was prespecified to reflect the clinically meaningful distinction between minimal nodal disease and established metastatic deposits.

To assess whether the extent of nodal tumor burden varied as an ordinal continuum across the three histopathological categories (ITC → micrometastasis → macrometastasis), primary tumor thickness was compared across groups using the Kruskal–Wallis test with the Jonckheere-Terpstra test for ordered alternatives. The association between primary tumor thickness and histological grade within the SLN-positive subgroup was evaluated using Spearman rank correlation. The multiplicity of prior interventions per patient was examined as a surrogate for lesion multiplicity and compared between groups using the Mann–Whitney U test.

Given the descriptive and hypothesis-generating nature of this study, no correction for multiple comparisons was applied; all reported *p*-values are nominal and should be interpreted accordingly. No formal power calculation was performed, as the study was designed to characterize a prospectively maintained consecutive institutional series rather than to test a pre-specified primary endpoint. Statistical analyses were performed using IBM SPSS Statistics (Version 26.0; IBM Corp., Armonk, NY, USA) and Python (Version 3.10) with the pandas, scipy, and statsmodels libraries.

## 3. Results

A total of 35 patients with clinically node-negative high-risk cutaneous squamous cell carcinoma underwent sentinel lymph node biopsy (SLNB) during the study period. The overall cohort had a median age of 70.0 years (IQR 62.0–76.0), and 26 patients (74.3%) were male. Median primary tumor diameter was 20.0 mm (IQR 15.0–28.0), and median tumor thickness was 5.0 mm (IQR 3.5–8.0). High histological grade (G ≥ 2) was present in 30 tumors (85.7%). Perineural invasion was documented in 3 patients (8.6%), immunosuppression in 4 (11.4%), and prior local recurrence in 4 patients (11.4%). Baseline clinicopathologic characteristics of the entire cohort and the two SLNB outcome subgroups are presented in Table 1, along with a summary of clinical follow-up duration.

Sentinel lymph node involvement was identified in 12 of 35 patients (34.3%). The number of sentinel nodes excised per patient ranged from 1 to 4 (median 2): 14 patients had a single sentinel node excised, 12 had 2 nodes, 8 had 3 nodes, and 1 patient had 4 nodes. All excised sentinel nodes from each patient were processed using the same ultrastaging protocol. SLN-positive patients had a median age of 71.5 years (IQR 63.0–75.2), and 9 (75.0%) were male. Compared with SLN-negative patients (*n* = 23), the SLN-positive group showed numerically larger median tumor diameter (23.0 mm vs. 18.0 mm) and greater median tumor thickness (5.5 mm vs. 4.5 mm), with a higher proportion of high-grade tumors (91.7% vs. 82.6%); however, these differences did not reach statistical significance given the sample size. Notably, perineural invasion was absent in all SLN-positive cases (0.0% vs. 13.0% in SLN-negative patients), and immunosuppression and prior recurrence were each present in only one SLN-positive patient (8.3%). Clinical follow-up data were available for all 35 patients. The median disease-free survival follow-up was 12 months (range 2–24 months) and the median overall survival follow-up was 16 months (range 3–24 months), corresponding to the study period from September 2008 to March 2010. No regional recurrence was identified during follow-up in any patient with ITC or micrometastatic nodal involvement. The single patient with macrometastatic disease died of disease at 9 months after SLNB. Nine patients in the overall cohort (25.7%) died during the follow-up period; causes of death were not limited to cSCC-related mortality and were not systematically adjudicated in this retrospective series.

**Figure 1:**
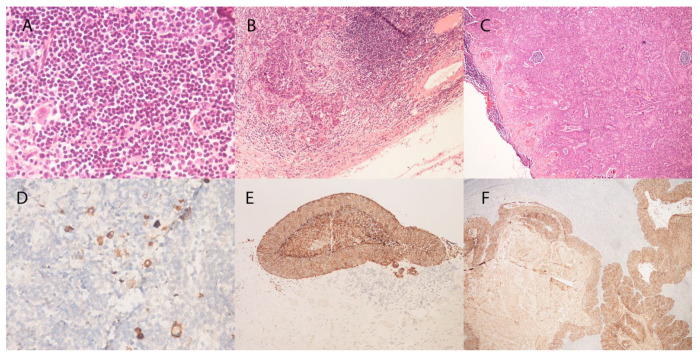
Representative histological appearances of nodal tumor-burden categories in sentinel lymph nodes of cSCC patients. (**A**) Isolated tumor cells (ITC): scattered individual cytokeratin-positive epithelial cells within the nodal parenchyma, not forming a cohesive cluster (H&E, ×40). (**B**) Micrometastasis: a cohesive epithelial deposit measuring between 0.2 mm and 2.0 mm in maximum diameter, with preserved nodal architecture in the surrounding parenchyma (H&E, ×10). (**C**) Macrometastasis: a large confluent tumor deposit exceeding 2.0 mm, replacing a substantial portion of the nodal architecture (H&E, ×4). (**D**–**F**) Corresponding sections immunostained with the pancytokeratin antibody AE1/AE3, confirming the epithelial (squamous) identity of the deposits in each tumor-burden category. Brown (DAB) chromogen; hematoxylin counterstain.

**Figure 2:**
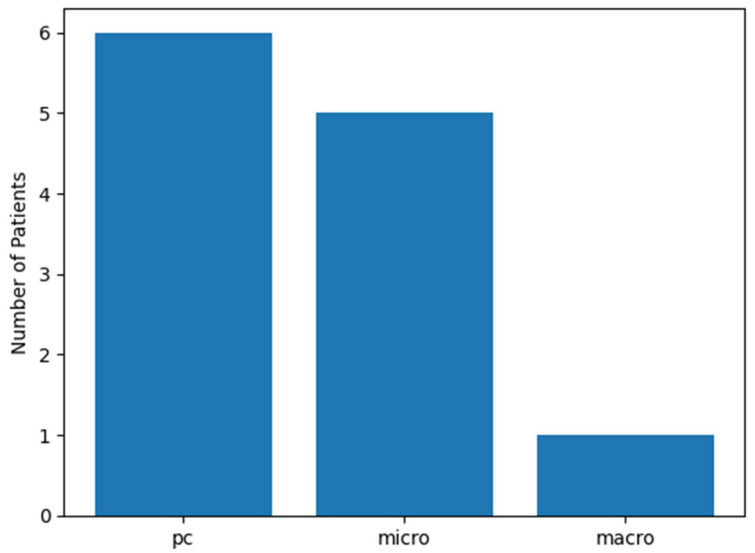
Distribution of sentinel lymph node tumor-burden patterns in SLN-positive cutaneous squamous cell carcinoma. Bar chart showing the relative frequency of isolated tumor cells (ITC/PC), micrometastases, and macrometastases among the 12 SLN-positive patients. ITC/PC accounted for 50% of positive cases, micrometastases for 41.7%, and macrometastasis for 8.3%. The predominance of minimal nodal disease highlights the histopathological heterogeneity of SLN-positive cSCC in clinically node-negative, high-risk patients.

Among the 12 SLN-positive patients, three histopathological patterns of nodal tumor burden were identified. Isolated tumor cells (ITC; also referred to as preclinical deposits [PC] in the context of cSCC) were identified in 6 patients (50.0%), micrometastases in 5 patients (41.7%), and macrometastasis in 1 patient (8.3%). Taken together, minimal nodal disease (ITC/PC) accounted for exactly half of all SLN-positive findings, while overt metastatic deposits (micrometastases and the single macrometastatic case combined) comprised the remaining half. Representative histological appearances of each nodal tumor-burden category—isolated tumor cells, micrometastases, and macrometastasis—are illustrated in Figure 1. The distribution of these tumor-burden categories is illustrated in Figure 2. This pattern underscores the predominance of minimal nodal involvement and confirms that SLN positivity in clinically node-negative high-risk cSCC is not a uniform histopathological entity.

For analytical purposes, patients were dichotomized into ITC/PC (*n* = 6) versus overt metastatic involvement (micrometastasis + macrometastasis combined; *n* = 6), given the limited sample size and the single macrometastatic case. Clinicopathologic characteristics of the two subgroups are presented in Table 2. No statistically significant difference was observed between ITC/PC and micro/macro groups for age (median 69.5 vs. 72.0 years; *p* = 0.688), primary tumor diameter (median 22.5 mm vs. 26.5 mm; *p* = 0.520), or histological grade (G ≥ 2 in 100% vs. 83.3%; *p* = 1.000). Sex distribution was similar (83.3% vs. 66.7% male; *p* = 1.000).

Tumor thickness showed a numerically notable and counterintuitive distribution between the two groups: median thickness was 8.0 mm (IQR 6.5–9.5) in ITC/PC patients and 4.0 mm (IQR 4.0–4.8) in those with micro/macrometastatic involvement, a difference that did not reach statistical significance (Mann–Whitney U; *p* = 0.293) but that represents a clinically and biologically meaningful reversal of the expected gradient. Perineural invasion, immunosuppression, and prior recurrence were absent or near-absent in both subgroups, precluding group-level comparisons for these variables.

**Figure 3:**
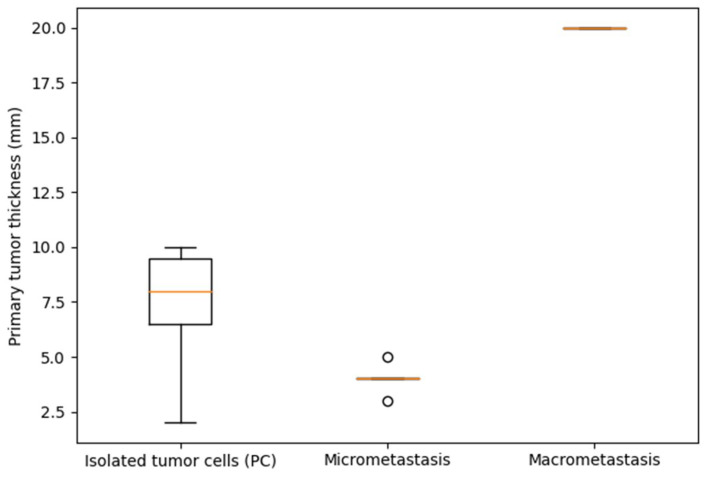
Primary tumor thickness according to sentinel lymph node tumor-burden pattern. Boxplot showing the distribution of primary tumor thickness (mm) stratified by SLN tumor-burden category (ITC/PC, micrometastasis, macrometastasis) among the 12 SLN-positive patients. Boxes represent the median and interquartile range; whiskers indicate the full range. The single macrometastatic case is displayed as an individual data point.

When the SLN-positive cohort was analyzed as an ordinal continuum across all three tumor-burden categories (ITC/PC → micrometastasis → macrometastasis), primary tumor thickness demonstrated a non-monotonic distribution: median thickness was 8.0 mm (IQR 6.5–9.5) in the ITC/PC group, 4.0 mm (IQR 4.0–4.0) in the micrometastatic group, and 20.0 mm in the single macrometastatic case. The overall Kruskal–Wallis test did not reach statistical significance (*p* = 0.065), but the numerical pattern is noteworthy: the group with the least nodal tumor burden (ITC/PC) carried the greatest median primary tumor thickness among patients with non-macrometastatic disease, contradicting a simple linear model of escalating primary tumor aggressiveness driving greater nodal colonization. The distribution of primary tumor thickness across nodal tumor-burden categories is illustrated in Figure 3. Despite not reaching statistical significance (Kruskal–Wallis *p* = 0.065), the non-monotonic distribution—with the ITC/PC group showing numerically greater median thickness than the micrometastatic group—argues against a simple linear relationship between primary tumor depth and extent of nodal colonization.

Within the SLN-positive cohort, Spearman rank correlation did not reveal a significant monotonic association between primary tumor thickness and histological grade (ρ = −0.136; *p* = 0.674), indicating that deeper tumors were not systematically more poorly differentiated in this subgroup. This internal dissociation between thickness and grade further supports the concept that, once nodal seeding has occurred, the primary tumor’s morphological phenotype does not reliably predict the volumetric extent of metastatic nodal involvement.

Multiplicity of surgical interventions, used as a surrogate marker for lesion multiplicity in this retrospective dataset, was also examined. The median number of interventions per patient did not differ significantly between SLN-positive and SLN-negative patients, nor between the ITC/PC and micro/macro subgroups within the SLN-positive cohort, indicating that the observed tumor-burden patterns were not confounded by a greater burden of multiple synchronous or metachronous lesions.

Collectively, these findings demonstrate that SLN positivity in this high-risk cSCC cohort is characterized by morphological heterogeneity, with a predominance of minimal nodal tumor burden. No clinicopathologic parameter derived from the primary tumor—including diameter, thickness, histological grade, perineural invasion, or lesion multiplicity—reliably stratified patients by extent of nodal involvement within the SLN-positive subset. These results indicate that SLN-positive cSCC encompasses biologically distinct presentations that are not captured by conventional primary tumor risk factors.

## 4. Discussion

In this consecutive institutional cohort of 35 patients with high-risk cutaneous squamous cell carcinoma (cSCC) who underwent sentinel lymph node biopsy (SLNB), nodal involvement was identified in 12 patients (34.3%). The principal contribution of the present study, however, is not the positivity rate per se, but the morphological characterization of nodal tumor burden within SLN-positive cases: isolated tumor cells (ITC/preclinical deposits) accounted for half of all positive findings (50%), micrometastases for 41.7%, and macrometastasis for only a single case (8.3%). This spectrum of minimal-to-overt nodal disease directly challenges the implicit assumption in many retrospective SLNB series that SLN positivity in cSCC constitutes a biologically uniform state.

The SLN positivity rate of 34.3% observed in our cohort is higher than most pooled estimates reported in the literature. A systematic review by Tejera-Vaquerizo et al., which analyzed 23 studies, found a pooled SLN positivity rate of 8% (95% CI, 5.1–10.8%) across unselected cSCC series [21]. A PRISMA-based meta-analysis of head and neck high-risk cSCC by Costantino et al. reported pooled SLN identification and positivity rates of 98.8% and 5.6%, respectively, further underscoring the wide variability in outcomes across institutions [11]. A systematic review and meta-analysis by Costantino et al., focusing specifically on high-risk HN cSCC, similarly documented substantial heterogeneity in positivity rates attributable to differences in patient risk stratification and pathological protocols [11]. This variability reflects at least three methodological confounders: (a) degree of patient selection, (b) anatomical distribution of primary tumors, and (c) pathological processing intensity. Our cohort was enriched for high-risk BWH-stage T2b tumors, and SLNB positivity has been reported at approximately 29.4% in prior work [22,23] for BWH T2b tumors. These considerations must be taken into account when comparing positivity rates across institutions, and our results should not be interpreted in isolation from this methodological context.

The choice of AE1/AE3 as the pancytokeratin marker warrants comment. AE1/AE3 was the institutional standard at the time of the study and remains the most widely used marker in published cSCC SLNB series. However, p40 offers greater squamous lineage specificity and would more definitively exclude non-squamous cytokeratin-positive cells. We recommend that future prospective studies consider incorporating p40 as a complementary or alternative marker for cSCC SLN analysis.

The wide variability in reported positivity rates underscores the central role of risk stratification in SLNB research. The Brigham and Women’s Hospital (BWH) staging system has been validated as superior to both the AJCC 7th and 8th editions in predicting poor outcomes, offering greater distinctiveness, homogeneity, and monotonicity of outcomes across stages [24,25]. BWH T2b/T3 tumors, comprising only 5% of most institutional cohorts, account for approximately 60% of poor outcomes, including 70% of nodal metastases and 83% of disease-specific deaths [22]. Additionally, BWH staging has been shown to outperform AJCC 8 in head and neck cSCC, with higher specificity (93%) and positive predictive value (30%) for identifying cases at risk for metastasis or death [25]. Given this context, the high positivity rate in our cohort is consistent with a risk-enriched population and supports the utility of BWH-based patient selection for SLNB.

Despite technical feasibility, the prognostic and therapeutic implications of SLNB in cSCC remain uncertain, as reflected in major guidelines. The American Academy of Dermatology (AAD) guideline explicitly states that the value of SLNB in cSCC remains unknown, noting that tumor size, thickness, angiolymphatic invasion, and perineural invasion have been proposed as risk factors for SLN positivity, but that small study sizes limit the assessment of prognostic parameters [26]. European consensus-based interdisciplinary guidelines (EDF/EADO/EORTC) similarly acknowledge the limited evidence and, in prior editions, did not recommend SLNB outside of a clinical trial context, though the most recent update emphasizes the emerging role of this procedure in carefully selected patients [27,28]. The lack of randomized data and the heterogeneity of published series make definitive recommendations premature at this time.

A key observation in our study is the predominance of minimal nodal tumor burden—ITC and micrometastases—among SLN-positive cases. This finding is directly dependent on the sensitivity of the pathological protocol employed. Our protocol included dual-tracer mapping with preoperative lymphoscintigraphy, intraoperative gamma-probe verification, size-adapted sectioning of lymph nodes, and selective pancytokeratin AE1/AE3 immunohistochemistry. Such ultrastaging protocols substantially increase the detection rate of small tumor deposits that would be missed by standard hematoxylin and eosin examination alone. Published series that do not employ immunohistochemistry or multiple-level sectioning will systematically underestimate the prevalence of ITC and small micrometastases [11,21]. Consequently, cohorts with rigorous ultrastaging protocols will shift the SLN-positive spectrum toward minimal disease—not because such patients harbor more aggressive disease, but because detection sensitivity is higher. This methodological effect is a critical confounder when interpreting and comparing positivity rates and tumor-burden distributions across studies.

The classification of nodal tumor burden into ITC, micrometastases, and macrometastases is well established in melanoma and breast cancer, with outcome-linked validation studies informing staging and treatment algorithms. In breast cancer, the prognostic distinction between ITC and micrometastases was formalized in the AJCC 2002/2010 staging updates, and large prospective registry studies have confirmed that ITC in sentinel nodes do not adversely affect disease-free or overall survival, whereas micrometastases show at most limited prognostic impact [18,29]. Notably, this distinction has proven clinically meaningful even across tumor types beyond breast cancer: in cervical carcinoma, a large multicenter analysis found that stratification of nodal metastases as ITC, micrometastases, or macrometastases carried no independent prognostic implication, highlighting that the clinical significance of tumor-burden classification is tumor-type specific and cannot be assumed to translate across histologies [30]. Similarly in melanoma, SLN tumor burden has been shown to correlate with non-sentinel lymph node involvement and disease-free survival, with submicrometastatic deposits (≤0.2 mm) carrying a distinctly more favorable prognosis than overt micro- or macrometastatic deposits [19,20,31]; this observation has directly informed treatment stratification in the era of adjuvant immunotherapy. In high-risk cSCC specifically, published SLNB series have begun to characterize positivity rates and technical outcomes, yet none have systematically reported nodal tumor burden by category [32]. Analogous outcome-linked validation of the ITC/micrometastasis/macrometastasis distinction in cSCC is entirely absent. Without such data, equating ITC/preclinical deposits with macrometastasis—or treating all SLN-positive cases as prognostically equivalent—is not evidence-based.

A noteworthy and biologically counterintuitive finding in our SLN-positive cohort was the relationship between primary tumor thickness and the extent of nodal tumor burden. Patients with ITC exhibited a numerically higher median primary tumor thickness (8.0 mm, IQR 6.5–9.5) than those with micrometastases (4.0 mm, IQR 4.0–4.0), with the single macrometastatic case measuring 20.0 mm. Although the overall comparison across three categories did not reach statistical significance (Kruskal–Wallis *p* = 0.065), the absence of a simple linear relationship between tumor depth and the extent of nodal colonization is conceptually important. Tumor thickness and histological grade are widely recognized as independent predictors of nodal metastasis in cSCC and are incorporated into staging frameworks [15,33]. Recurrence, perineural invasion, lymphovascular invasion, and tumor size ≥ 2 cm have also been significantly associated with lymph node metastasis in multivariate analyses [34]. However, these factors predict the binary event of “metastasis yes or no”—they are not guaranteed to predict the volumetric extent of nodal colonization once metastasis has already occurred. Our data suggest that, within the conditioned subgroup of SLN-positive patients, primary tumor characteristics do not reliably stratify the magnitude of nodal tumor burden. This is consistent with the concept that early lymphatic seeding in cSCC may be a partially stochastic event that does not require a threshold of primary tumor bulk.

The prognostic significance of SLN status itself in cSCC remains contested in the literature. Major guidelines and large cohort studies have identified histological risk factors for poor outcomes but have not established a consistent survival advantage associated with SLN-directed management [6,26]. A systematic review and meta-analysis of high-risk head and neck cSCC by Costantino et al. confirmed technical reliability of the procedure, with an SLN identification rate exceeding 95%, but the available data were insufficient to demonstrate an independent outcome benefit from SLNB-directed management [11]. Similarly, the systematic review by Tejera-Vaquerizo et al. found no studies reporting on predictors of SLN involvement after adjustment for confounders, underscoring the methodological limitations of the existing literature [21]. In this context, our finding that minimal nodal disease predominates among SLN-positive cases raises important questions: if ITC and micrometastases do not carry the same prognostic weight as macrometastases, the apparent prognostic heterogeneity in SLN-positive cSCC series may, in part, reflect the mixing of biologically distinct disease states under a single binary classification.

From a dermatopathological standpoint, our results provide a strong argument for structured reporting of SLN tumor-burden categories in cSCC. The ITC/micrometastasis/macrometastasis classification, already embedded in the TNM systems for breast cancer and melanoma, should be systematically applied and reported in cSCC SLNB specimens. Where feasible, additional morphological information—including deposit distribution (subcapsular versus parenchymal), multifocality, architectural effacement, and extracapsular extension—should accompany the categorical classification. Without standardized tumor-burden reporting, cross-study comparisons will remain confounded by both patient selection and variability in pathological processing, and meta-analyses assessing the prognostic significance of minimal disease in cSCC will remain underpowered.

The strengths of this study include a consecutive institutional cohort design, a standardized dual-tracer mapping protocol with intraoperative gamma-probe verification, and a pathological processing approach optimized to detect small nodal deposits. Its principal limitations are the small overall cohort (*n* = 35) and particularly limited SLN-positive subgroup (*n* = 12, including only a single macrometastatic case), which preclude multivariable modeling and render within-subgroup comparisons underpowered; the selective rather than universal application of pancytokeratin immunohistochemistry; the limited follow-up duration (median 12 months DFS; range 2–24 months), which was insufficient to capture cSCC-specific mortality events across tumor-burden subgroups; and the retrospective single-center design, which limits generalizability. Accordingly, all reported *p*-values are nominal and all within-subgroup comparisons should be interpreted as descriptive and hypothesis-generating.

## 5. Conclusions

SLN positivity in high-risk cSCC is morphologically heterogeneous, with minimal nodal disease (ITC and micrometastases) accounting for the majority of positive cases. No primary tumor feature reliably stratified the extent of nodal colonization within SLN-positive patients. Structured tumor-burden reporting should be adopted as standard practice, and adequately powered prospective studies are needed to establish the prognostic relevance of each category.

## Figures and Tables

**Table 1:** Clinicopathologic characteristics of the study cohort, stratified by sentinel lymph node (SLN) status.

Variable	All Patients (*n* = 35)	SLN-Positive (*n* = 12)	SLN-Negative (*n* = 23)	*P*-Value
Demographics				
Age (years), median (IQR)	70.0 (62.0–76.0)	71.5 (63.0–75.2)	69.0 (61.5–77.0)	0.724
Male sex, *n* (%)	26 (74.3%)	9 (75.0%)	17 (73.9%)	1.000
Primary tumor characteristics				
Tumor diameter (mm), median (IQR)	20.0 (15.0–28.0)	23.0 (17.2–30.5)	18.0 (13.5–25.0)	0.228
Tumor thickness (mm), median (IQR)	5.0 (3.5–8.0)	5.5 (4.0–8.5)	4.5 (3.0–7.5)	0.368
High grade (G ≥ 2), *n* (%)	30 (85.7%)	11 (91.7%)	19 (82.6%)	0.641
Perineural invasion, *n* (%)	3 (8.6%)	0 (0.0%)	3 (13.0%)	0.540
Immunosuppression, *n* (%)	4 (11.4%)	1 (8.3%)	3 (13.0%)	1.000
Prior recurrence, *n* (%)	4 (11.4%)	1 (8.3%)	3 (13.0%)	1.000
Clinical follow-up				
Disease-free follow-up (months), median (IQR)	12.0 (10.0–20.0)	11.5 (9.8–16.8)	12.0 (11.0–21.0)	0.349
Overall survival follow-up (months), median (IQR)	16.0 (11.0–21.0)	11.5 (9.8–16.8)	17.0 (12.0–21.0)	0.070

Abbreviations: IQR, interquartile range; SLN, sentinel lymph node; G, histological grade. *p*-values: Mann–Whitney U test for continuous variables; Fisher’s exact test for categorical variables. Follow-up duration *p*-values reflect comparability of observation times between groups, not differences in outcomes. Note: No regional recurrence was identified in any ITC or micrometastatic case during follow-up. The single macrometastatic patient died of disease at 9 months.

**Table 2:** Clinicopathologic Characteristics of SLN-Positive Patients Stratified by Nodal Tumor-Burden Pattern.

Variable	ITC/PC (*n* = 6)	Micro/Macro (*n* = 6)	*p*-Value
Age (years), median (IQR)	69.5 (63.5–74.8)	72.0 (64.5–75.8)	0.688
Male sex, *n* (%)	5 (83.3%)	4 (66.7%)	1.000
Tumor diameter (mm), median (IQR)	22.5 (18.5–28.8)	26.5 (16.5–34.2)	0.520
Tumor thickness (mm), median (IQR)	8.0 (6.5–9.5)	4.0 (4.0–4.8)	0.293
High grade (G ≥ 2), *n* (%)	6/6 (100%)	5/6 (83.3%)	1.000
Perineural invasion, *n* (%)	0 (0%)	0 (0%)	–
Immunosuppression, *n* (%)	0 (0%)	1 (16.7%)	1.000
Recurrence (prior), *n* (%)	1 (16.7%)	0 (0%)	1.000

## Data Availability

The data that support the findings of this study are available upon request from the corresponding author.
